# Spanish Validation of the Problem Area in Diabetes-Pediatric Version Survey and Its Weak Association with Metabolic Control Parameters in Pediatric Diabetes: A Cross-Sectional Multicenter Study

**DOI:** 10.3390/jcm14020523

**Published:** 2025-01-15

**Authors:** Elisa Civitani Monzón, María Pilar Ferrer Duce, Antonio De Arriba Muñoz, Irune Goicoechea Manterola, Rosa Yelmo Valverde, Josep-Oriol Casanovas-Marsal

**Affiliations:** 1 Miguel Servet University Hospital, Avenida Isabel la Católica 1-3, 50009 Zaragoza, Aragón, Spain; ecivitani@salud.aragon.es (E.C.M.); mpferrerd@salud.aragon.es (M.P.F.D.); adearriba@salud.aragon.es (A.D.A.M.); 2 Instituto de Investigación Sanitaria Aragón, Avenida San Juan Bosco 13, 50009 Zaragoza, Aragón, Spain; 3 Sant Joan de Déu Hospital, Passeig Sant Joan de Déu 2, 08950 Barcelona, Catalonia, Spain; irune.goicoechea@sjd.es; 4 Ramon y Cajal University Hospital, Carretera Colmenar Viejo km. 9100, 28034 Madrid, Comunidad de Madrid, Spain; rosa.yelmo@salud.madrid.org

**Keywords:** type 1 diabetes mellitus, diabetes distress, adolescent, psychological

## Abstract

**Background**: Type 1 diabetes mellitus (T1DM) in pediatric patients often leads to emotional distress, impacting self-management. The PAID-Peds survey measures diabetes-related emotional burden but lacks a validated Spanish version. This study aimed to validate the Spanish PAID-Peds survey in children and adolescents with T1DM and correlate it with diabetic metabolic control parameters. **Methods**: A cross-sectional study was conducted from October 2022 to December 2023, recruiting 636 patients aged 8–17 years from three Spanish hospitals. Psychometric properties were assessed using Cronbach’s alpha for reliability and confirmatory factor analysis for construct validity. Associations between PAID-Peds scores and clinical measures, such as HbA1c, were examined. **Results**: The final sample consisted of 538 participants (84.59% response rate). The PAID-Peds survey showed high internal consistency (Cronbach’s alpha = 0.90). The confirmatory factor analysis indicated a satisfactory model fit (χ^2^ = 812.28, *p* < 0.001; RMSEA = 0.08). Weak correlations were found between PAID-Peds scores and HbA1c (r = 0.14, *p* < 0.001). **Conclusions**: The Spanish PAID-Peds survey is a reliable tool for assessing emotional burden in pediatric T1DM patients. Integrating it into clinical practice may improve early identification of emotional distress, aiding in better diabetes management. Further research should explore its application over time and in intervention studies.

## 1. Introduction

Type 1 diabetes mellitus (T1DM) is one of the most common endocrine diseases in childhood [1]. In 2021, an estimated 651,700 children and adolescents under 15 years were living with the condition worldwide, a figure that is expected to increase by 3–4% annually over the following decade [1,2]. Managing T1DM in children requires ongoing decisions related to insulin dosing, dietary adherence, and blood glucose monitoring to prevent both immediate and long-term complications [3]. The Diabetes Control and Complications Trial (DCCT) found that maintaining blood glucose within the target range is more challenging for adolescents compared to adults, highlighting the complexity of managing the condition during this developmental stage.

Effective treatment of T1DM relies on strict self-care practices [4] to achieve optimal blood glucose levels and maintain a good quality of life. The International Society for Pediatric and Adolescent Diabetes (ISPAD) recommends a target glycosylated hemoglobin (HbA1c) level of less than 7%. However, achieving this target can be challenging due to the complex daily routines involved, with less than 25% of young patients reaching this goal [5,6].

The self-management of T1DM is time-consuming and disrupts daily routines for both patients and their families [4]. This burden falls on the patients themselves and their caregivers and can have a substantial psychosocial impact, negatively affecting quality of life [7].

“Diabetes distress”, a term introduced in the 1990s, refers to the emotional strain caused by managing diabetes and its self-care. It affects 20–40% of patients, including feelings of worry and fear, but is distinct from depression as it centers on stress associated with the demands of the condition [6]. This aligns with data from Spanish-speaking populations, where 36% of children and adolescents exhibit psychological difficulties within the first year, predominantly depression and anxiety, compared to non-diabetic youth [8]. Addressing these emotional concerns is crucial, as adolescence often brings challenges such as psychological vulnerability, behavioral changes, body image concerns, and family conflicts [4]. These factors not only increase the emotional burden but also interfere with metabolic control, making it harder to achieve glycemic targets during this period [9].

Psychosocial stressors such as anxiety and depression can lead to inconsistent self-management behaviors, including poor adherence to insulin regimens, irregular blood glucose monitoring, and unhealthy eating patterns. These behaviors, in turn, contribute to suboptimal metabolic control, creating a vicious cycle that exacerbates both the psychological and physiological burden of T1DM [10]. Integrating psychosocial care into diabetes management plans has been shown to improve both emotional well-being and glycemic control, underscoring the importance of addressing these factors holistically [11]. Educational interventions have proven effective in reducing the emotional burden of diabetes [11], and major international organizations such as ISPAD recommend structured educational programs during adolescence.

The complexity of diabetes management calls for validated and standardized scales to assess patient-reported outcomes (PROMs) [12], providing a more holistic approach to care [13,14].

The Problem Area in Diabetes (PAID, File S1) questionnaire is one of the most widely used tools to assess diabetes distress in adults [15]. Although other tools exist for measuring emotional distress in children with diabetes, such as the PedsQL [16] and the Diabetes Stress Scale (DSS) [17], these focus more on general quality of life or stress rather than specifically addressing the emotional issues related to diabetes management. In contrast, PAID-Peds was specifically designed to assess emotional distress associated with diabetes in children, making it an especially relevant choice for our study population. PAID-Peds also includes a version for parents (PAID-PR) [18], reinforcing a holistic approach to diabetes treatment that incorporates the family. Its cultural adaptability, validity, and focus on the emotional impact of diabetes justify its use in this study, as it provides a direct measure of the daily emotional challenges faced by children with diabetes.

Despite the availability of other tools, such as the Appraisal of Diabetes Scale (ADS) [19], the Audit of Diabetes-Dependent Quality of Life (ADDQOL) [20], and the Diabetes Health Profile (DHP) [21], these instruments do not specifically address the emotional distress associated with diabetes in children. Moreover, while these tools have been adapted and validated in various languages, including Turkish [22], German [23], and Danish [24], no version of PAID-Peds is currently available for Spanish-speaking children aged 8–17 years. The results obtained from PAID-Peds^®^ can be effectively used to improve the clinical management of pediatric patients with type 1 diabetes. For example, high scores on the scale can identify patients experiencing greater emotional burden related to diabetes management, which could justify psychological intervention or support from a multidisciplinary team. In these cases, an integrated approach combining both medical and psychological care may be crucial to reduce the stress associated with the condition, which in turn may improve treatment adherence and metabolic control. Our study aims to fill this gap by validating the Spanish translation of the PAID-Peds and correlating it with metabolic control parameters.

## 2. Methods

### 2.1. Study Design, Setting, and Participants

A cross-sectional, observational, multicenter study involving 538 patients aged 8–17 years diagnosed with T1DM, who were undergoing treatment and follow-up at the Miguel Servet University Hospital (Zaragoza, Spain), Ramón y Cajal University Hospital (Madrid, Spain), and Sant Joan de Déu Hospital (Barcelona, Spain), was conducted from 1 October 2022 to 31 December 2023.

### 2.2. Instrument with Validity and Reliability

The PAID-Peds^®^ survey was designed to assess the emotional health of children with T1DM in the pediatric population aged 8–17 years. Originally developed in English by Markowitz et al., this instrument specifically measures “youth-reported burden related to type 1 diabetes management” [25]. PAID-Peds^®^ is a 20-item structured, self-administered tool. Participants responded on a five-point Likert scale, with 0 being “agree”, 2 “neither agree nor disagree”, and 4 “disagree”. The total score is obtained by reversing the scores of each item—with the original response value of 0 being 4, 1 being 3, 2 being 2, 3 being 1, and 4 being 0—and averaging all the items. For a 100-point scale, the results must be multiplied by 25. Scores closer to 100 indicate a higher risk of loss of emotional health. The survey was validated with an internal consistency of 0.94 (Cronbach’s alpha) and an intraclass correlation coefficient of 0.66 (*p* < 0.001). Patients with scores higher than 41 are considered to be suffering from “emotional burnout” [26]. The discrepancy between the adult and pediatric PAID scoring methods lies in how the final score is calculated. In the adult version, the scores for each item are summed and then multiplied by 1.25, resulting in a maximum score of 100 points. In contrast, in the pediatric version, the scores are averaged first and then multiplied by 25, also yielding a maximum score of 100 points. Thus, while both methods lead to the same maximum score, the difference lies in that the pediatric method averages the responses before applying the multiplication factor, whereas the adult method sums them first, which may better account for the variability in children’s responses.

To use the survey, the study authors contacted Markowitz et al. and the Pediatric, Adolescent, and Young Adult Section at the Joslin Diabetes Center (Department of Psychiatry, Harvard Medical School, Boston, MA, USA), who authorized the translation of the instrument into Spanish and subsequent validation.

**Figure 1:**
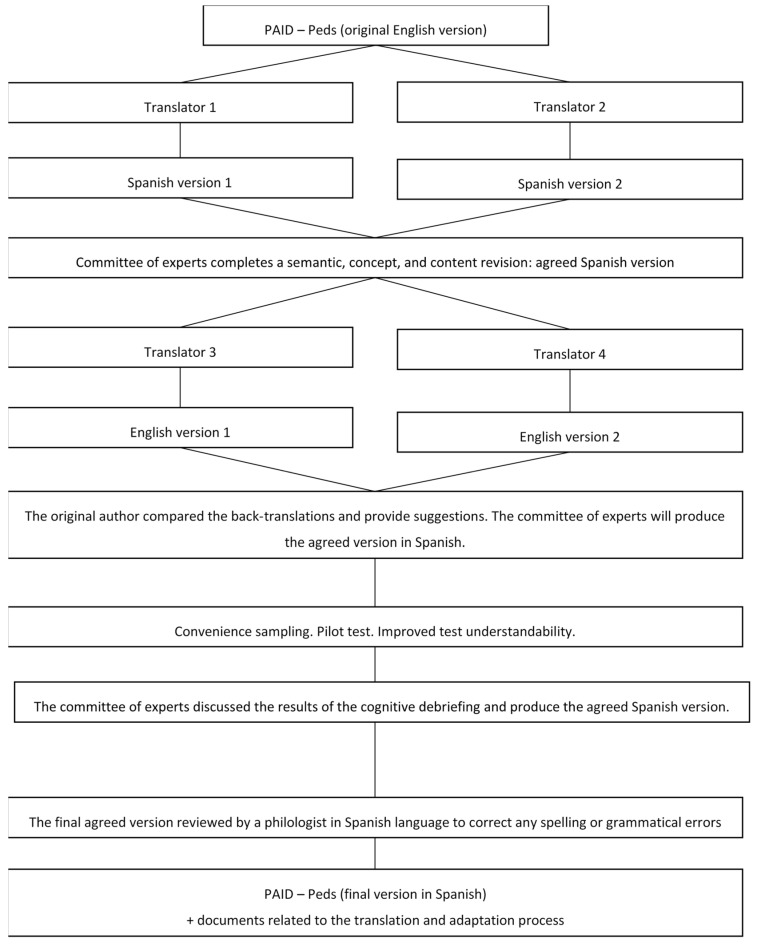
Stages of translation into Spanish, cultural adaptation, and validation of PAID-Peds^®^ survey (Casanovas-Marsal et al. [28]).

The study protocol for translation and cultural adaptation was then designed [27] and the Spanish version was validated [28] through qualitative validation and a pilot study involving 30 patients from the Miguel Servet University Hospital (Zaragoza, Spain), the Ramón y Cajal University Hospital (Madrid, Spain), and the Sant Joan de Déu Hospital (Barcelona, Spain), and we followed an eight-step structured approach aligned with the principles of good practice outlined by the International Society for Pharmacoeconomics and Outcomes Research Task Force for Translation and Cultural Adaptation [29] (Figure 1).

Step 1: Forward translation. Two professional translators, both native Spanish speakers, independently translated the questionnaire into Spanish. Step 2: Reconciliation and synthesis. A panel of five multidisciplinary experts in diabetes and endocrinology compared and merged the two translations into a single unified version. Step 3: Back-translation. Two professional translators, both native English speakers, independently translated the questionnaire back into English. Step 4: Comparison and harmonization. The back-translations were compared to the original English version, and any discrepancies were resolved to ensure equivalence. Step 5: Cognitive debriefing. A multicenter pilot study was conducted with 30 young patients diagnosed with T1DM, selected through consecutive sampling, to assess the comprehensibility and relevance of the Spanish version of the PAID-Peds^®^ survey. Step 6: Review of cognitive debriefing results. The feedback from the cognitive debriefing phase was reviewed and incorporated to refine the translation. Step 7: Proofreading, spelling, and grammar revision. The final version of the translated questionnaire was thoroughly proofread for spelling, grammar, and linguistic accuracy. Step 8: Final report. A comprehensive report was prepared detailing the translation and adaptation process (Table 1).

### 2.3. Sampling and Recruitment

**Figure 2:**
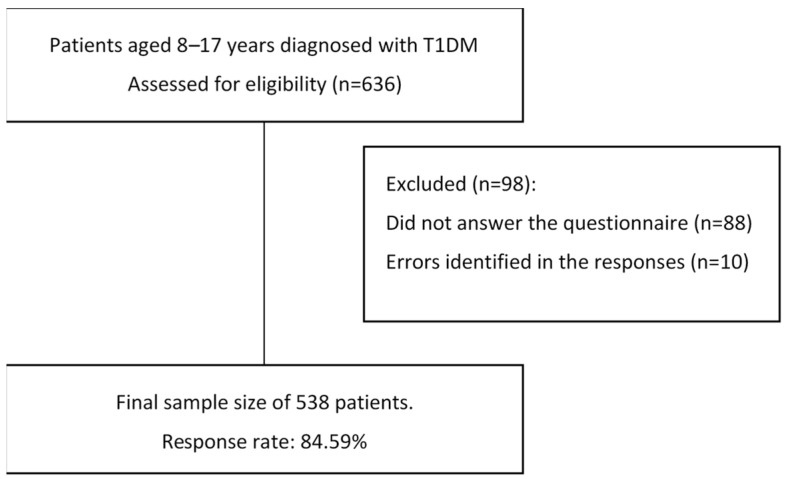
Flow diagram of participation.

Initially, 636 patients aged 8–17 years were diagnosed with T1DM (as per ISPAD criteria) at least one year prior. All patients undergoing treatment and follow-up at the Miguel Servet University Hospital (Zaragoza, Spain), the Ramón y Cajal University Hospital (Madrid, Spain), and the Sant Joan de Déu Hospital (Barcelona, Spain) from 1 October 2022 to 31 December 2023 were invited to participate in the study. Of these, 88 did not respond, and questionnaires from 10 additional patients contained errors, resulting in a final sample size of 538 (response rate: 84.59%) (Figure 2).

### 2.4. Data Sources/Collection

Upon patient inclusion, the nurses at the Department of Educational Nursing in Pediatric Diabetes of each participating hospital explained the details of the study to the patients and their guardians. This included providing a participant information sheet outlining the study’s objectives. With regard to the ethics of participation, after receiving written and verbal explanations, parents/legal guardians and children aged 12 or older voluntarily agreed to participate by signing informed consent forms. The sociodemographic variables were as follows: sex (male, female), age (years), and type of family (two-parent, single-parent, adoptive, separated, compound, and extended). The clinical variables were as follows: weight; SD weight; height; SD height; body mass index [30]; systolic and diastolic blood pressure; chronic complications; HbA1c (%) at 3, 6, 9, and 12 months prior to the study; glucose management indicator; glucose in range downloaded directly from the CGM sensor; year of disease onset; insulin administration type; number of hospitalizations due to ketoacidosis in the previous year; number of episodes of hyperglycemia treated in the emergency department; history of hypoglycemia with and without loss of consciousness; hypoglycemia with loss of consciousness and administration of glucagon; hypoglycemia without loss of consciousness requiring immediate medical attention; hypoglycemia without loss of consciousness and with hospitalization; other autoimmune disorders; and time taken to complete the survey.

### 2.5. Data Analysis

Data analysis was performed using Jamovi^®^ 2.1.23. Qualitative variables were presented using the frequency distribution of the percentages of each category. Quantitative variables were studied using the goodness-of-fit to a normal distribution (Kolmogorov–Smirnov test), indicating the central tendency (mean) and dispersion (standard deviation). The association between variables was determined using Student’s *t*-test (normal distribution) and the Mann–Whitney U-test (non-parametric distribution) when one variable was quantitative, and the chi-squared test when both variables were qualitative. Moreover, Fisher’s exact test was used if the frequencies observed were lower than 5. The association between the variables studied was explored using hypothesis contrast tests, with comparisons of proportions when both variables were qualitative (chi-squared, Fisher’s exact test), comparisons of means when one variable was quantitative (Student’s *t*-test, ANOVA, or the Mann–Whitney U or Kruskall–Wallis test if they did not follow a normal distribution), and bivariate correlations (Pearson’s correlation coefficient) when both variables were quantitative. Effects were considered significant at *p* < 0.05. Cronbach’s alpha was calculated to determine the reliability of test (considered to be good for values higher than 0.8) [31].

The adaptation and validity of the survey was checked by means of a confirmatory factor analysis. The difference between the model and the data observed was assessed using the chi-squared test of exact fit (considered optimal for values with *p* > 0.05). The chi-squared test is used to compare observed and expected frequencies to assess how well a model fits the data, especially when dealing with categorical data or testing the goodness of fit between a theoretical model and observed data. Other goodness-of-fit measures were also used, such as the comparative fit index (CFI; values higher than 0.80 indicate a good fit) [32], the Tucker–Lewis Index (TLI; values equal to or greater than 0.8 are considered acceptable) [33], the Standardized Root Mean Square Residual test (SRMR; values lower than 0.08 are considered to be indicative of a good fit [34]), and the Root Mean Square Error of Approximation test (RMSEA; values of up to 0.08 are considered to be acceptable) [35,36]. Although CFI and TLI values greater than 0.90 are generally considered indicative of good model fit in modern psychometric research, values between 0.80 and 0.90 are still widely accepted as indicating an acceptable fit, particularly in studies where the focus is on evaluating a single model, rather than comparing multiple competing models. The selection of CFI and TLI in this analysis was based on their suitability for assessing the fit of confirmatory factor models, particularly in the context of questionnaire validation. These indices are well recognized for evaluating model fit in psychometric research, especially when comparing the proposed model against a null baseline model. Given that the main focus of this study was on assessing the absolute fit of a single proposed model, without the need to compare it with alternative models, the values obtained for CFI and TLI were considered sufficient for the objectives of the study.

### 2.6. Ethical Considerations

Confidentiality of participant data was maintained throughout the study. All collected data were coded and pseudonymized. Data collection adhered to Spanish data protection legislation (Organic Law 3/2018, of December 5th, on Personal Data Protection and Guarantee of Digital Rights). The study followed national and international ethical guidelines, including the Declaration of Helsinki. Furthermore, the study ensured compliance with the European Association for Children in Hospital Charter, approved by the European Parliament in 1986. This charter upholds children’s right to be accompanied, which aligns with the study’s commitment to promoting a humane approach to care. All participants in the study were informed through the participant information sheet, and if they decided to participate, they signed the informed consent form. To ensure the comprehension of the survey content among younger participants, especially those between 8 and 10 years old, assistance during the survey was implemented as part of the instrument adaptation and validation process. In some cases, younger participants were supported by an adult or facilitator (such as a parent or a member of the research team) to clarify any doubts about the questions. However, it was ensured that the facilitators did not influence the responses. The study was approved by the Research Ethics Committee of the Autonomous Community of Aragon, of the Ramón y Cajal University Hospital of Madrid (C.P.-C.I. PI21/00425), and of the Fundación Sant Joan de Déu (C.I. PIC-33-22).

## 3. Results

### 3.1. Demographic and Clinical Characteristics of Participants

A total of 538 patients (84.59%) answered the survey correctly. Of these, 278 (51.67%) were male and 260 (48.33%) were female. The mean age of T1DM onset was 7.59 ± 3.73 years in females and 7.41 ± 3.94 years in males (sociodemographic and metabolic control data, score on survey, and response time for the study population are available in Table 2 and Table 3, and Figure 1 and Figure 2). Of all the participants, 54 (10.04%) belonged to single-parent families, 412 (76.58%) to two-parent families, four (0.74%) were adopted, and in 68 (12.64%) cases the parents had separated.

### 3.2. Incidence of Acute Descompensations, Therapeutic Regimen, and Medical Background

The therapeutic regimen used by 220 (40.89%) of the subjects was continuous subcutaneous insulin infusion (CSII), with the remaining 318 (59.11%) patients receiving multiple daily injections (MDI) of insulin.

Regarding medical background, 40 (7.43%) had been diagnosed with celiac disease, 26 (4.83%) with autoimmune thyroiditis, five (0.93%) with multiple endocrinopathy, one (0.19%) with diabetic retinopathy, two (0.37%) with diabetic nephropathy, and one (0.19%) with diabetic neuropathy.

With regard to the occurrence of acute decompensations in the past year, 7.62% presented at least one DKA, 6.88% hyperglycemia requiring emergency care, and 2.79% hypoglycemia with loss of consciousness and administration of glucagon.

### 3.3. Survey Score, Response Time, Correlations, and Multivariate Regression Analysis of PAID-Peds and Metabolic Control Parameters

The mean overall score obtained in the PAID-Peds^®^ survey (Annex 1) was 45.05 ± 18.13 and the mean time to answer the survey was 4.22 ± 2.78 min. Table 4 shows the metabolic control data and PAID-Peds score survey classified by age group.

Weak correlations were observed between the total PAID-Peds score and HbA1c (r = 0.14, *p* < 0.001) and time in the range of 70–180 mg/dL (r = −0.11, *p* < 0.01) and hyperglycemia >250 mg/dL (r = 0.10, *p* < 0.02).

In the case of the glucose range < 54 mg/dL and 54–70 mg/dL, no correlations were found with any item on the questionnaire. However, for the observed range between 180–250 mg/dL, correlations were found with item 2 (0.12; *p*: 0.006) and item 14 (0.18; *p* < 0.001). For the range > 250 mg/dL, correlations were found with item 2 (0.13; *p* = 0.002), item 3 (0.17; *p* ≤ 0.001), item 5 (0.12; *p* = 0.005), item 7 (0.13; *p* = 0.001), item 10 (0.12; *p* = 0.004), item 13 (0.09; *p* = 0.03), and item 14 (0.21; *p* < 0.001).

In the multivariate linear regression analysis, a significant association was identified between the total PAID-Peds score and HbA1c levels, after adjusting for glucose, coefficient of variation, and GMI (estimate: 2.6102; *p* = 0.048). Subsequently, an extended model was developed, including the variable glucose categorized into specific ranges. The results were as follows: for glucose range <54 mg/dL: estimate = 2.578, *p* = 0.048; for glucose range 54–70 mg/dL: estimate = 2.725, *p* = 0.036; for glucose range 70–180 mg/dL: estimate = 2.751, *p* = 0.034; for glucose range 180–250 mg/dL: estimate = 2.729, *p* = 0.0356; and for glucose range >250 mg/dL: estimate = 2.742, *p* = 0.034.

By sex, the mean age for males when answering the survey was 13.61 ± 2.78 years (95% CI: 13.28–13.94), the mean response time was 4.40 ± 2.55 min (95% CI: 4.10–4.71), and the mean overall score obtained in the PAID-Peds^®^ survey was 43.08 ± 17.78 (95% CI: 40.99–45.18). For females, the corresponding values were 3.59 ± 2.71 years (95% CI: 13.26–13.92), 4.02 ± 2.99 min (95% CI: 3.65–4.38), and 47.15 ± 18.30 (95% CI: 44.92–49.39). According to these findings, significant differences were found between sex and time required to answer the survey (*p* < 0.001) and the overall score obtained in the PAID-Peds^®^ survey (*p* < 0.001). Females required less time to complete the survey (*p* < 0.001) than males and obtained a higher overall score (*p* < 0.001). The frequency distribution for the percentages (%) for each answer for each item in the PAID-Peds^®^ survey is available on Table 5. In the multivariate linear regression analysis, there were no significance differences between PAID-Peds score and the metabolic control parameters adjusting by sex.

No association was found between the age at which the survey was answered and the score for the group of patients as a whole (r = −0.04, *p* = 0.35). However, when stratifying by sex, males presented a negative correlation with the overall PAID-Peds^®^ score (r = −0.14, *p* = 0.02).

Negative correlations were found between the time required to answer the questionnaire (r = −0.28; *p* < 0.0001) and the time since onset of diabetes (r = −0.10; *p* < 0.01).

When considering the type of therapy used (multiple insulin doses or continuous infusion insulin pump), no significant differences were found between these two types of administration and the overall score obtained in the survey (43.62 ± 18.09 vs. 46.04 ± 18.12; *p* = 0.13) or the response time (4.36 ± 2.86 vs. 4.12 ± 2.72; *p* = 0.16).

The overall PAID-Peds^®^ score obtained was not correlated with either the time since onset of diabetes, current age, or time required to answer the survey.

### 3.4. Response Distribution for Survey Items and Sex

The aspects with which both sexes were in greatest agreement (“agree”) were, in the following order, statement Q20, “My parents worry about me and my diabetes too much” (34.39%, *n* = 185), followed by Q12, “My friends and/or family act like the ’diabetes police’ (for example, always reminding me to eat right, check blood sugars, or take insulin)” (24.26%, *n* = 130), Q9, “I am annoyed when I have to stop what I am doing to check my blood sugar” (22.86%, *n* = 123), Q6, “I feel upset when my blood sugar is out of range” (22.12%, *n* = 119), Q4, “It bothers me to think so much about what I eat” (21.56%, *n* = 116), and Q19, “I worry about going low, especially during physical activities (for example, sports, playing outside, dance class)” (21%, *n* = 113). The remaining statements achieved a much lower degree of agreement (<14%).

The statements exhibiting the greatest disagreement (“disagree”) among participants (more than 58% and a difference of more than 20 points with respect to the others) were Q11, “I feel embarrassed about having diabetes”, and Q15, “I feel like I do not fit in with other kids/teens my age because of my diabetes”. The statements exhibiting the lowest degree of disagreement (“disagree”) were those that exhibited the greatest agreement (“agree”).

No statement presented the highest degree of agreement (“agree”) or disagreement (“disagree”) for all participants.

No participant obtained a score of 0 points on the survey and only two obtained a score of more than 90 points.

Significant differences were found for both sexes for the statements Q1, “I feel sad a lot when I think about having diabetes”, Q8, “I feel left out when I cannot eat things other kids/teens are eating”, and Q13, “I am tired of remembering to give insulin shots or bolus”, based on the response values for each item in the survey (Table 6).

No differences were found between the time of evolution of diabetes and the results for each item in the survey.

### 3.5. Reliability Analysis and Model Fit

The Cronbach’s alpha obtained in the reliability analysis was 0.90 and the range of correlations for each item was 0.30–0.69 (Table 7). Similarly, the result of the confirmatory factor analysis, the chi-squared test of exact fit of the Spanish model to the PAID-Peds^®^ survey, was *p* = 0.00 (X2: 812.28; gl: 170). In the confirmatory factor analysis, the comparative fit index (CFI) yielded a value of 0.83, and the Tucker–Lewis Index (TLI) was 0.81, both suggesting an acceptable model fit. Additionally, the Standardized Root Mean Square Residual (SRMR) was 0.06, and the Root Mean Square Error of Approximation (RMSEA) exceeded 0.08, indicating a good model fit according to these criteria.

## 4. Discussion

The aim of this study was to validate the psychometric properties of the PAID-Peds^®^ questionnaire in Spanish by way of a cross-sectional, multicenter study.

Diabetes-related distress (DRD) is common among individuals with type 1 diabetes, with prevalence rates ranging from 18–45%, including adolescents [37]. DRD has been shown to correlate with elevated HbA1c levels [5,38]. In our study, the mean HbA1c value of our sample was 7.34 ± 0.99%, which slightly exceeds the target range recommended by the ISPAD guidelines [39]. Additionally, the mean time spent in the target glucose range (70–180 mg/dL) was 61.33%, which is below the recommended 70% [10,39]. Notably, only one third of patients managed to stay within this range for more than 70% of the time.

Our findings also highlight a positive outcome, as the majority of children and adolescents met the recommended standard for hypoglycemia, with means levels for level 1 and 2 hypoglycemia falling below the 4% target [10,39]. However, a significant issue observed was the prolonged time spent in hyperglycemia, specifically above 250 mg/dL. Approximately 30.8% of patients adhered to the recommended time in this range (less than 5% of the time), while 69.2% exceeded the recommended time above 250 mg/dL (11.95% observed vs. 5% recommended) [10,38]. Additionally, 60.97% of patients adhered to the recommended standard for moderate hyperglycemia (180–250 mg/dL, less than 25% of the time), reflecting moderate control, though still below the optimal level [10,39].

Future studies should explore the impact of continuous glucose monitoring systems on these metrics.

It is essential to achieve good metabolic control, especially during adolescence, to avoid future complications, and the HbA1c value in this group was 7.45%. Given the unique circumstances of this life stage, which involves both hormonal and psychological changes, it is often more difficult to avoid out-of-range values and, as such, these patients have a higher risk of complications such as DKA and hospitalization, as reported in various studies [40]. Our study only shows a weakly positive correlation between the PAID-Peds^®^ survey and level 1 hypoglycemia.

Despite the absence of significant differences in PAID-Peds^®^ scores between patients using different therapies (CSII or MDI), this result aligns with the findings of Vesco et al. [41], the first study to examine the use of technology and diabetes-related distress. In both cases, all patients utilized a technological device, specifically continuous glucose monitoring (CGM), which indeed represents a significant difference compared to adolescents who do not use such devices. Significant differences were observed in statements Q2, Q3, and Q5, which relate to emotional impacts of diabetes, as well as in Q10, “I am tired of trying to figure out my insulin dose at every meal”, and Q16, “I am annoyed by having to rotate injection sites or pump infusion sites”. The latter two statements pertain to technical self-management skills, which are more common in patients using MDI. This is because integrated systems provide algorithms that simplify dose calculation, and the rotation of infusion cannulas in pumps does not require daily adjustments. For these statements, patients using MDI were more worried than those using automated insulin pump systems, which would support the idea that technology is helping to reduce the mental burden resulting from having diabetes [42].

As in the original survey [25], no significant differences were found in scores based on age. However, as was the case, the results were supported by the findings of the narrative review on 25 years of diabetes distress (DD) research [6] and other research studies differences were found between sex and test scores, with women achieving higher values [43]. The question that achieved the highest agreement for both sexes (over a third of the sample) was Q20: “My parents worry about me and my diabetes too much”. The burden of living with diabetes, which in this case is shared with other family members involved in caring for the patient due to their age, implies a high psychosocial impact on the family unit. The response to this question seems to indicate that many children and teenagers are fully aware of this situation.

Statement Q6, “I feel upset when my blood sugar is out of range”, was given a score of 0 or 1 by 68.40% of subjects of both sexes, thus indicating the importance for them of achieving glucose levels within the target range of other aspects related to the injections, diet, or social aspects. This aspect achieved the lowest disagreement of all.

In contrast, the greatest disagreement was obtained for Q11, “I feel embarrassed about having diabetes”, for females (26.95%), and Q15, “I feel like I do not fit in with other kids/teens my age because of my age because of my diabetes”, for males (32.71%). In females, this appears to be more closely related to how they perceive themselves, whereas in males it is related to the worry about being accepted by their peers. The fact that Q15 was selected most by subjects with the longest time of evolution since diagnosis supports the idea that they want to feel equal to everyone else over time.

Although the PAID-Peds^®^ survey was developed without establishing a cut-off point for diabetes-related distress, PAID and its various versions have been widely studied in the past few years [26] in an attempt to establish a cut-off point that helps healthcare professionals to identify the emotional burden caused by diabetes. Overall, 40.15% of the sample obtained a score of less than 40 points, 51.11% in the range 40–70 points, and 8.74% >70 points. In other words, over half of the children and teenagers in the study obtained scores greater than 40 points [26] (66.45% aged more than 13 years), which may indicate the presence of a moderate emotional burden. With regard to the score obtained in the PAID-Peds^®^ survey, there were no significant differences as regards the maturity of the female cohort, whereas males tended to present higher scores at younger ages. It was also found that males needed a longer time to complete the questionnaire.

Diabetes management in children and young people raises very specific needs for the different stages of development, differing family situations, and biological and psychological changes [10]. These developmental stages are critical as they influence not only the child’s physical health but also their psychosocial well-being. The various changes—such as puberty, cognitive development, and shifting family dynamics—can present challenges in maintaining optimal diabetes management. Family support systems, social influences, and the child’s ability to understand and manage their condition evolve during this period, further emphasizing the need for personalized care strategies.

All these aspects influence health outcomes [6] and increase the risk of worsening of the blood glucose levels, diabetes-related distress, and quality of life [38]. As such, pediatric diabetes teams should be able to evaluate the educational and psychosocial factors that negatively affect the ability to achieve satisfactory self-management. It is therefore necessary to include validated and specially adapted instruments in the care routines for young T1DM patients [44] that allow patient-reported outcome measures (PROMs) to be measured [14]. The evaluation and early detection of the emotional distress caused by diabetes will help to implement more efficient strategies and interventions in those patients requiring them.

Although the Spanish version of the PAID-Peds^®^ survey has proven to be a valid and reliable tool for identifying diabetes-related emotional distress in pediatric patients, its integration into routine diabetes care is crucial for maximizing its clinical impact. To implement this tool effectively, healthcare professionals should receive training on how to incorporate the PAID-Peds^®^ survey as part of regular follow-up care for children and adolescents with type 1 diabetes. Institutions should consider facilitating online access to the survey, reducing administrative burdens and allowing for easier implementation in clinical settings. Additionally, incorporating the Spanish PAID-Peds^®^ survey into electronic health record systems would enable healthcare providers to track emotional distress levels in real time, offering an opportunity for early intervention and targeted psychosocial support. By embedding this tool into standard care practices, professionals will be better equipped to identify distress early and tailor interventions that improve both psychological and clinical outcomes for young patients with diabetes.

The strengths of this study include its multicenter design, which enhances the generalizability of the findings across different clinical settings. Additionally, the validation of the Spanish version of the PAID-Peds^®^ questionnaire contributes to the availability of a reliable tool for assessing diabetes-related distress in Spanish-speaking pediatric populations. However, the cross-sectional nature of the study limits the ability to establish causal inferences. Although the sample encompasses a wide range of ages, disease durations, and treatment regimens, it may not fully represent the heterogeneity of the broader pediatric type 1 diabetes population. Furthermore, the reliance on self-reported data could introduce recall or social desirability biases. Lastly, the absence of a longitudinal follow-up prevents the assessment of long-term changes in distress levels or the impact of interventions. Despite the weak associations between metabolic parameters and PAID-Peds^®^ scores, the tool remains valuable for assessing psychosocial distress in pediatric diabetes management. This highlights the importance of PAID-Peds^®^ in evaluating emotional and psychological factors, beyond its potential link to metabolic control.

Future research could focus on the longitudinal evaluation of diabetes-related distress in the pediatric population using the Spanish version of the PAID-Peds^®^ questionnaire. A long-term follow-up would allow for the examination of how emotional distress levels evolve over time, particularly during different stages of adolescence and in relation to therapeutic interventions, such as the use of continuous glucose monitoring (CGM) technologies. Additionally, further studies should explore the effectiveness of psychological interventions aimed at reducing diabetes-related distress, assessing their impact on both psychological health and clinical outcomes, such as HbA1c levels and time in glucose range. This research direction builds on the findings of the study and opens up the possibility of exploring how emotional factors and interventions may influence diabetes management in children and adolescents over time.

In conclusion, the Spanish version of the PAID-Peds^®^ questionnaire has proven to be a valid and reliable tool for assessing diabetes-related distress in pediatric patients with type 1 diabetes. The findings highlight the significant emotional burden experienced by many children and adolescents, underlining the importance of addressing psychological well-being as part of comprehensive diabetes care.

## Figures and Tables

**Table 1:** Qualitative results of the translation, back-translation, and cultural adaptation of the PAID-Peds questionnaire from English to Spanish.

Original	Translation 1 Version 1	Translation 2 Version 2	Reconciliation and Synthesis	Back-Translation 3 Version 1	Back-Translation 4 Version 2	Final Report Revised by Hispanic Philologist
Problem Areas in Diabetes-Pediatric (Paid-Peds) Survey	Áreas Problemáticas en la Diabetes (Paid)-Cuestionario Pediátrico (Paid-Peds)	Áreas Problemáticas en la Diabetes-Cuestionario Pediátrico (Paid-Peds)	Áreas Problemáticas en la Diabetes (Paid)-Cuestionario Pediátrico (Paid-Peds)	Problem Areas in Diabetes (Paid)-Pediatric Questionnaire (Paid-Peds)	Problem Areas in Diabetes (Paid)-Paediatric (Paid-Peds) Survey	Aspectos Problemáticos de la Diabetes (Paid)-Cuestionario Pediátrico (Paid-Peds)
The following statements describe diabetes-related issues that may or may not be a concern for you. For each item, choose the ONE answer that best describes how much you agree or disagree with that statement.	Las siguientes afirmaciones describen cuestiones relacionadas con la diabetes que pueden suponer un problema para ti o no. Elige la respuesta que mejor describa tu grado de acuerdo o desacuerdo con la afirmación de cada ítem.	Las siguientes afirmaciones describen aspectos relacionados con la diabetes que pueden preocuparte o no. En cada ítem, elige la respuesta que describe mejor tu nivel de acuerdo o desacuerdo con la afirmación.	Las siguientes afirmaciones describen cuestiones relacionadas con la diabetes que pueden suponer un problema para ti o no. Elige la respuesta que mejor describa tu grado de acuerdo o desacuerdo con la afirmación de cada ítem.	The following statements describe aspects related to diabetes that may or may not represent a problem for you. Choose the answer that best describes your degree of agreement or disagreement with the statement for each item.	The following statements describe diabetes-related issues that may or may not be a problem for you. Choose the answer that best describes your degree of agreement or disagreement with the statement in each item.	Las siguientes afirmaciones describen cuestiones relacionadas con la diabetes que pueden suponer o no un problema para ti. Elige la respuesta que mejor describa tu grado de acuerdo o desacuerdo con la afirmación de cada punto.
1. I feel sad a lot when I think about having diabetes.	1. Me pongo muy triste cuando pienso que tengo diabetes.	1. Me entristece mucho pensar que tengo diabetes.	1. Me pongo muy triste cuando pienso que tengo diabetes.	1. I get very sad when I think I have diabetes.	1. I feel very sad when I think about having diabetes.	1. Me pongo muy triste cuando pienso en que tengo diabetes.
2. I feel like diabetes has taken over my life.	2. Tengo la sensación de que la diabetes controla mi vida.	2. Siento como si la diabetes hubiera tomado el control de mi vida.	2. Siento que la diabetes controla mi vida.	2. I feel that diabetes controls my life.	2. I feel like diabetes has taken control of my life.	2. Siento que la diabetes controla mi vida.
3. I feel like it is my fault when my blood sugar is out of range.	3. Siento que es mi culpa cuando mi nivel de glucosa está fuera de rango.	3. Siento que es mi culpa cuando mi azúcar en sangre está fuera de los límites normales.	3. Siento que es mi culpa cuando mi nivel de azúcar está fuera de rango.	3. I feel that it is my fault when my blood sugar is out of range.	3. I feel it is my fault when my blood sugar is out of range.	3. Siento que es mi culpa cuando mi nivel de azúcar está fuera de rango.
4. It bothers me to think so much about what I eat.	4. Me molesta pensar tanto lo que como.	4. Me fastidia tener que estar pensando lo que como.	4. Me molesta tener que estar pensando lo que como.	4. It annoys me to have to think about what I eat.	4. It bothers me to have to think about what I eat.	4. Me molesta tener que estar pensando en lo que como.
5. I worry all the time about how diabetes will affect me when I am older.	5. Me preocupa todo el tiempo cómo me afectará la diabetes cuando sea mayor.	5. Me preocupo constantemente por cómo me afectará la diabetes de mayor.	5. Me preocupo constantemente por cómo me afectará la Diabetes cuando sea mayor.	5. I am always worrying about how diabetes will affect me when I am older.	5. I worry all the time about how diabetes will affect me when I am older.	5. Me preocupo constantemente por cómo me afectará la diabetes cuando sea mayor.
6. I feel upset when my blood sugar is out of range.	6. Me desagrada que mi nivel de glucosa esté fuera de rango.	6. Me molesta que mi azúcar en sangre esté fuera de los límites normales.	6. Me molesta que mi nivel de glucosa esté fuera de rango.	6. It annoys me that my blood sugar is out of range.	6. It bothers me that my blood sugar is out of range.	6. Me molesta que mi nivel de azúcar esté fuera de rango.
7. I am too tired of having diabetes to take care of it.	7. Estoy demasiado cansado/a de tener diabetes para cuidar de la enfermedad.	7. Estoy demasiado cansado/a de tener diabetes para cuidar de ella.	7. Estoy demasiado cansado/a de tener diabetes para cuidar de ella.	7. I am too tired of having diabetes to care about it.	7. I am too tired of having diabetes to take care of it.	7. Estoy demasiado cansado/a de tener diabetes como para cuidar de ella.
8. I feel left out when I cannot eat things other kids/teens are eating.	8. Me siento excluido/a cuando no puedo comer lo que comen otros niños/niñas/adolescentes.	8. Me siento excluido/a cuando no puedo comer lo mismo que otros niños/as o adolescentes.	8. Me siento excluido/a cuando no puedo comer lo mismo que otros niños/as o adolescentes.	8. I feel excluded when I cannot eat the same as other children or teenagers.	8. I feel excluded when I cannot eat what other kids/teenagers eat.	8. Me siento excluido/a cuando no puedo comer lo mismo que otros niños/as o adolescentes.
9. I am annoyed when I have to stop what I am doing to check my blood sugar.	9. Me fastidia tener que dejar de hacer algo para comprobar mi nivel de glucosa.	9. Me desagrada tener que dejar de hacer lo que estoy haciendo para medir mi azúcar en sangre.	9. Me fastidia tener que dejar de hacer lo que estoy haciendo para medir mi azúcar en sangre.	9. It bothers me that I have to stop doing what I am doing to measure my blood sugar.	9. It annoys me to have to stop what I am doing to check my blood sugar.	9. Me fastidia tener que dejar de hacer lo que estoy haciendo para medir mi azúcar en sangre.
10. I am tired of trying to figure out my insulin dose at every meal.	10. Estoy cansado/a de intentar calcular la dosis de insulina en cada comida.	10. Estoy cansado/a de intentar calcular la dosis de insulina en cada comida.	10. Estoy cansado/a de intentar calcular la dosis de insulina en cada comida.	10. I am tired of trying to calculate the insulin dose at each meal.	10. I am tired of trying to calculate my insulin dose at every meal.	10. Estoy cansado/a de intentar calcular la dosis de insulina en cada comida.
11. I feel embarrassed about having diabetes.	11. Me avergüenzo de tener diabetes.	11. Me avergüenza tener diabetes.	11. Me siento avergonzado de tener diabetes.	11. I feel ashamed of having diabetes.	11. I feel embarrassed about having diabetes.	11. Me avergüenza tener diabetes.
12. My friends and/or family act like the “diabetes police” (for example, always reminding me to eat right, check blood sugars, or take insulin).	12. Mis amigos y/o familia actúan como la «policía de la diabetes» (siempre recordándome comer bien, comprobar el nivel de glucosa o ponerme la insulina).	12. Mis amigos y/o mi familia se comportan como la «policía de la diabetes» (por ejemplo, me recuerdan continuamente que coma bien, que me mida el azúcar en sangre o que me ponga insulina).	12. Mis amigos y/o mi familia se comportan como la «policía de la diabetes» (por ejemplo, me recuerdan continuamente que coma bien, que me mida el azúcar en sangre o que me ponga insulina.	12. My friends and/or family behave like the “diabetes police” (for example, they are always reminding me to eat correctly, to measure my blood glucose, or to take insulin).	12. My friends and/or family act like the “diabetes police” (for example, they remind me all the time to eat well, to check my blood sugar, or to take my insulin).	12. Mis amigos y/o mi familia se comportan como la «policía de la diabetes» (por ejemplo, me recuerdan continuamente que coma bien, que me mida el azúcar en sangre o que me ponga la insulina).
13. I am tired of remembering to give insulin or shots or bolus.	13. Estoy cansado/a de tener que recordar ponerme insulina o bolos.	13. Estoy cansado/a de acordarme de las inyecciones de insulina o de ponerme bolos.	13. Estoy cansado/a de tener que recordar ponerme las inyecciones de insulina o los bolos.	13. I am tired of having to remember to inject my insulin or bolus.	13. I am tired of having to remember my insulin shoots or bolus.	13. Estoy cansado/a de tener que acordarme de ponerme las inyecciones de insulina o los bolos.
14. It seems like no matter how hard I try, my blood sugars are out of control.	14. Tengo la sensación de que, haga lo que haga, mis niveles de glucosa siempre están fuera de control.	14. Parece que, por mucho que me esfuerce, mi azúcar en sangre siempre está fuera de control.	14. Tengo la sensación de que, haga lo que haga, mis niveles de azúcar siempre están fuera de control.	14. I feel that, whatever I do, my blood sugar is always out of control.	14. I feel like, no matter what, my blood sugar is always out of range.	14. Tengo la sensación de que, haga lo que haga, mis niveles de azúcar siempre están fuera de control.
15. I feel like I do not fit in with other kids/teens my age because of my diabetes.	15. Siento que no encajo con otros niños/niñas/adolescentes de mi edad debido a la diabetes.	15. Siento que no encajo con otros niños/as o adolescentes de mi edad por culpa de la diabetes.	15. Siento que no encajo con otros niños/as o adolescentes de mi edad por culpa de la diabetes.	15. I feel that I do not fit in with other children or teenagers of my age because of diabetes.	15. I feel like I do not fit in with other kids/teenagers my age due to my diabetes.	15. Siento que no encajo con otros niños/as o adolescentes de mi edad por culpa de la diabetes.
16. I am annoyed by having to rotate injection sites or pump infusion sites.	16. Me fastidia tener que rotar las zonas de inyección o de infusión de la bomba.	16. Me desagrada tener que cambiar las áreas de punción o de infusión de la bomba.	16. Me molesta tener que rotar las zonas de inyección o de infusión de la bomba.	16. It annoys me to have to change the injection or pump infusion zones.	16. It bothers me to have to rotate injection or pump infusion areas.	16. Me molesta tener que rotar las zonas de inyección o de infusión de la bomba.
17. I feel angry a lot when I think about having diabetes.	17. Me enfado mucho cuando pienso en que tengo diabetes.	17. Me enfado mucho al pensar que tengo diabetes.	17. Me enfado mucho cuando pienso en que tengo diabetes.	17. I get very angry when I think I have diabetes.	17. I feel very angry when I think about having diabetes.	17. Me enfado mucho cuando pienso en que tengo diabetes.
18. My friends and family do not understand what it is like to have diabetes.	18. Mis amigos y familia no entienden lo que es tener diabetes.	18. Mis amigos y mi familia no entienden cómo es tener diabetes.	18. Mis amigos y familia no entienden lo que es tener diabetes.	18. My friends and family do not understand what it is like to have diabetes.	18. My friends and family do not understand what it is like to have diabetes.	18. Mis amigos y mi familia no entienden lo que es tener diabetes.
19. I worry about going low, especially during physical activities (for example, spots, playing outside, dance class).	19. Me preocupa tener una bajada, especialmente al hacer ejercicio (por ejemplo, al practicar deportes, jugar en el exterior o en clase de baile).	19. Me preocupa tener una bajada de azúcar, especialmente durante las actividades físicas (deportes, juegos en el exterior, clases de baile, etc.).	19. Me preocupa tener una bajada de azúcar, especialmente durante el ejercicio (deportes, juegos en el exterior, clases de baile, etc.).	19. I worry about having a decrease in blood sugar levels, especially during exercise (sports, outdoor games, dance classes, etc.).	19. I worry about having a drop in blood sugar, especially during physical exercise (sports, outdoor games, dance class, etc.).	19. Me preocupa tener una bajada de azúcar, especialmente al hacer ejercicio (deportes, juegos al aire libre, clases de baile, etc.).
20. My parents worry about me and my diabetes too much.	20. Mis padres se preocupan demasiado por mí y por la diabetes.	20. Mis padres se preocupan demasiado por mí y mi diabetes.	20. Mis padres se preocupan demasiado por mí y por mi diabetes.	20. My parents worry too much about me and my diabetes.	20. My parents worry too much about me and my diabetes.	20. Mis padres se preocupan demasiado por mí y por mi diabetes.

**Table 2:** Sociodemographic and metabolic control data, score on survey, and response time for the study population (T1DM: diabetes mellitus type 1; kg: kilograms; SD: standard deviation; cm: centimeters; BMI: body mass index; SBP: systolic blood pressure; DBP: diastolic blood pressure; HbA1c: glycated hemoglobin; GMI: glucose management indicator; CV: coefficient of variation; IQR: interquartile range).

	Mean	SD	CI 95%		Median	IQR
			Lower	Upper		
**Current Age (years)**	13.60	2.74	13.37	13.83	13.89	4.36
Age onset (years)	7.49	3.84	7.17	7.82	7.61	5.95
Time with T1DM (years)	6.16	3.92	5.83	6.50	5.57	5.60
Weight (kg)	52.73	16.15	51.40	54.14	52.60	22.48
SD weight	0.07	0.98	−0.01	0.16	−0.09	1.12
Height (cm)	158.17	14.74	156.92	159.42	160.00	20.03
SD height	0.11	1.07	0.02	0.20	0.07	1.51
BMI	20.60	3.84	20.27	20.92	20.27	4.82
SBP (mmHg)	112.57	12.84	111.48	113.66	112.00	15.00
DBP (mmHg)	69.38	8.94	68.61	70.13	70.00	11.00
Hb1Ac (%)	7.34	0.99	7.26	7.43	7.20	1.20
Hb1Ac previous 3 months (%)	7.31	0.92	7.23	7.39	7.20	1.10
Hb1Ac previous 6 months (%)	7.30	1.01	7.22	7.39	7.20	1.00
Hb1Ac previous 9 months (%)	7.23	0.99	7.15	7.32	7.10	1.20
Hb1Ac previous 12 months (%)	7.25	1.03	7.16	7.34	7.10	1.10
Current GMI (%)	7.29	0.80	7.22	7.36	7.20	0.90
Glucose (mg/dL)	165.21	30.69	162.62	167.81	160.50	39.00
CV diabetes (%)	37.75	7.53	37.11	38.39	37.35	9.43
Time <54 mg/dL (%)	0.57	1.28	0.46	0.68	0.00	1.00
Time 54–70 mg/dL (%)	3.36	4.87	2.95	3.77	2.00	3.00
Time 70–180 mg/dL (%)	61.33	16.64	59.92	62.74	63.00	24.75
Time 180–250 mg/dL	22.79	8.93	22.04	23.55	22.00	12.00
Time >250 mg/dL (%)	11.95	11.52	10.97	12.92	9.00	15.00
Time spent answering the questionnaire (min)	4.22	2.78	3.98	4.45	3.48	2.74
PAID-Peds^®^ total score	45.05	18.13	43.52	46.59	45.00	27.05

**Table 3:** Percentage of patients adhering to the standard percentage for glucose within the target range, categorized by blood glucose range.

Glucose Range	Recommendations	% Patients
<54 mg/dL	<1%	68.22
54–70 mg/dL	<4%	64.68
70–180 mg/dL	>70%	33.09
180–250 mg/dL	<25%	60.97
>250 mg/dL	<5%	30.86

**Table 4:** Metabolic control data and PAID-Peds score survey by age group (SD: standard deviation; HbA1c: glycated hemoglobin; GMI: glucose management indicator; CV: coefficient of variation).

	Age Group (Years Old)	N	Mean	SD	*p*
Hb1Ac (%)	8–12	161	7.07	0.76	<0.001
	13–18	377	7.46	1.06	
Current GMI (%)	8–12	161	7.13	0.62	<0.001
	13–18	377	7.36	0.86	
Glucose (mg/dL)	8–12	161	158.54	27.64	<0.001
	13–18	377	168.06	31.50	
CV diabetes (%)	8–12	161	36.59	6.28	0.03
	13–18	377	38.24	7.96	
Time <54 mg/dL (%)	8–12	161	0.36	0.70	0.04
	13–18	377	0.66	1.46	
Time 54–70 mg/dL (%)	8–12	161	3.06	4.39	0.26
	13–18	377	3.49	5.06	
Time 70–180 mg/dL (%)	8–12	161	65.03	14.73	<0.001
	13–18	377	59.75	17.17	
Time 180–250 mg/dL	8–12	161	22.12	8.80	0.14
	13–18	377	23.08	8.98	
Time >250 mg/dL (%)	8–12	161	9.43	9.81	<0.001
	13–18	377	13.02	12.03	
Time spent answering the questionnaire (min)	8–12	161	5.32	3.29	<0.001
	13–18	377	3.75	2.38	
PAID-Peds^®^ total score	8–12	161	46.39	17.83	0.29
	13–18	377	44.48	18.26	

**Table 5:** Frequency distribution for the percentages (%) for each answer for each item in the PAID-Peds^®^ survey (*n* = 538).

Item % (*n*)	Agree	->	Neither Agree Nor Disagree	->	Disagree
Q1	6.51 (35)	15.24 (82)	29.18 (157)	20.63 (111)	28.44 (153)
Q2	10.22 (55)	20.26 (109)	25.28 (136)	26.39 (142)	17.84 (96)
Q3	10.59 (57)	30.48 (164)	29.37 (158)	16.73 (90)	12.83 (69)
Q4	21.56 (116)	25.28 (136)	17.84 (96)	22.86 (123)	12.45 (67)
Q5	10.59 (57)	23.23 (125)	27.32 (147)	19.52 (105)	19.33 (104)
Q6	22.12 (119)	46.28 (249)	19.70 (106)	8.36 (45)	3.53 (19)
Q7	9.85 (53)	13.20 (71)	19.52 (105)	28.62 (154)	28.81 (155)
Q8	10.97 (59)	19.52 (105)	13.20 (71)	22.49 (121)	33.83 (182)
Q9	22.86 (123)	28.25 (152)	17.29 (93)	19.33 (104)	12.27 (66)
Q10	9.67 (52)	14.50 (78)	26.77 (144)	28.07 (151)	21.00 (113)
Q11	2.79 (15)	5.39 (29)	10.59 (57)	22.68 (122)	58.55 (315)
Q12	24.16 (130)	30.11 (162)	26.39 (142)	13.01 (70)	6.32 (34)
Q13	12.27 (66)	20.45 (110)	22.86 (123)	23.98 (129)	20.45 (110)
Q14	7.06 (38)	13.38 (72)	23.05 (124)	31.97 (172)	24.54 (132)
Q15	2.79 (15)	3.35 (18)	9.67 (52)	26.02 (140)	58.18 (313)
Q16	13.94 (75)	17.29 (93)	21.38 (115)	24.54 (132)	22.86 (123)
Q17	6.51 (35)	13.94 (75)	18.96 (102)	25.46 (137)	35.13 (189)
Q18	7.99 (43)	13.94 (75)	21.19 (114)	21.75 (117)	35.13 (189)
Q19	21.00 (113)	35.50 (191)	21.19 (114)	13.20 (71)	9.11 (49)
Q20	34.39 (185)	32.16 (173)	21.19 (114)	7.99 (43)	4.28 (23)

**Table 6:** Results from the total score of PAID-Peds^®^ survey for each response item by sex.

% (*n*)	0		1		2		3		4		*p* Value (CC)
	Female	Male	Female	Male	Female	Male	Female	Male	Female	Male	CC
Q1	3.7	2.8	8.6	6.7	17.1	12.1	7.8	12.8	11.2	17.3	<0.001
	(20)	(15)	(46)	(36)	(92)	(65)	(42)	(69)	(60)	(93)	0.19
Q2	5.4	4.8	11.5	8.7	11.9	13.4	12.3	14.1	7.2	10.6	0.19
	(29)	(26)	(62)	(47)	(64)	(72)	(66)	(76)	(39)	(57)	N/A
Q3	5.58	5.02	16.17	14.31	13.20	16.17	7.25	9.48	6.13	6.69	0.47
	(30)	(27)	(87)	(77)	(71)	(87)	(39)	(51)	(33)	(36)	N/A
Q4	11.52	10.04	13.57	11.71	8.18	9.67	10.04	12.83	5.02	7.43	0.22
	(62)	(54)	(73)	(63)	(44)	(52)	(54)	(69)	(27)	(40)	N/A
Q5	5.39	5.20	11.90	11.34	13.20	14.13	9.29	10.22	8.55	10.78	0.86
	(29)	(28)	(64)	(61)	(71)	(76)	(50)	(55)	(46)	(58)	N/A
Q6	11.52	10.59	23.23	23.05	8.55	11.15	3.16	5.20	1.86	1.67	0.38
	(62)	(57)	(125)	(124)	(46)	(60)	(17)	(28)	(10)	(9)	N/A
Q7	5.58	4.28	6.69	6.51	9.67	9.85	14.50	14.13	11.90	16.91	0.28
	(30)	(23)	(36)	(35)	(52)	(53)	(78)	(76)	(64)	(91)	N/A
Q8	6.13	4.83	11.15	8.36	6.51	6.69	11.15	11.34	13.38	20.45	0.04
	(33)	(26)	(60)	(45)	(35)	(36)	(60)	(61)	(72)	(110)	0.14
Q9	11.34	11.52	14.31	13.94	7.43	9.85	8.92	10.41	6.32	5.95	0.75
	(61)	(62)	(77)	(75)	(40)	(53)	(48)	(56)	(34)	(32)	N/A
Q10	4.46	5.20	8.55	5.95	12.64	14.13	13.01	15.06	9.67	11.34	0.38
	(24)	(28)	(46)	(32)	(68)	(76)	(70)	(81)	(52)	(61)	N/A
Q11	1.86	0.93	3.53	1.86	5.95	4.65	10.04	12.64	26.95	31.60	0.08
	(10)	(5)	(19)	(10)	(32)	(25)	(54)	(68)	(145)	(170)	N/A
Q12	12.27	11.90	13.94	16.17	12.64	13.75	5.20	7.81	4.28	2.04	0.11
	(66)	(64)	(75)	(87)	(68)	(74)	(28)	(42)	(23)	(11)	N/A
Q13	6.51	5.76	12.08	8.36	8.92	13.94	10.78	13.20	10.04	10.41	0.03
	(35)	(31)	(65)	(45)	(48)	(75)	(58)	(71)	(54)	(56)	0.14
Q14	4.09	2.97	7.43	5.95	11.71	11.34	14.13	17.84	10.97	13.57	0.28
	(22)	(16)	(40)	(32)	(63)	(61)	(76)	(96)	(59)	(73)	N/A
Q15	1.49	1.30	1.86	1.49	5.58	4.09	13.94	12.08	25.46	32.71	0.16
	(8)	(7)	(10)	(8)	(30)	(22)	(75)	(65)	(137)	(176)	N/A
Q16	6.13	7.81	8.18	9.11	12.27	9.11	12.45	12.08	9.29	13.57	0.11
	(33)	(42)	(44)	(49)	(66)	(49)	(67)	(65)	(50)	(73)	N/A
Q17	3.53	2.97	6.69	7.25	10.04	8.92	13.38	12.08	14.68	20.45	0.23
	(19)	(16)	(36)	(39)	(54)	(48)	(72)	(65)	(79)	(110)	N/A
Q18	4.83	3.16	7.43	6.51	10.59	10.59	9.11	12.64	16.36	18.77	0.23
	(26)	(17)	(40)	(35)	(57)	(57)	(49)	(68)	(88)	(101)	N/A
Q19	9.29	11.71	18.40	17.10	10.97	10.22	5.58	7.62	4.09	5.02	0.48
	(50)	(63)	(99)	(92)	(59)	(55)	(30)	(41)	(22)	(27)	N/A
Q20	17.29	17.10	15.61	16.54	9.48	11.71	3.90	4.09	2.04	2.23	0.93
	(93)	(92)	(84)	(89)	(51)	(63)	(21)	(22)	(11)	(12)	N/A

**Table 7:** Test correlations and Cronbach’s alpha for each question in the PAID-Peds^®^ survey, Spanish version.

Item Reliability Statistics	Item–Rest Correlation	If Item Dropped
		Cronbach’s α
Q1	0.66	0.89
Q2	0.61	0.89
Q3	0.39	0.90
Q4	0.68	0.89
Q5	0.51	0.90
Q6	0.30	0.90
Q7	0.65	0.89
Q8	0.60	0.89
Q9	0.57	0.90
Q10	0.65	0.89
Q11	0.50	0.90
Q12	0.46	0.90
Q13	0.69	0.89
Q14	0.52	0.90
Q15	0.57	0.90
Q16	0.37	0.90
Q17	0.71	0.89
Q18	0.42	0.90
Q19	0.36	0.90
Q20	0.37	0.90

## Data Availability

The data presented in this study are available upon request due to privacy or ethical restrictions. Requests for the data can be made to the corresponding author.

## Supplementary Materials

The following supporting information can be downloaded at: https://www.mdpi.com/article/10.3390/jcm14020523/s1, File S1: APPENDIX 2: Problematic aspects of diabetes—pediatric questionnaire.
