# Supplementary material for: Spanish Validation of the Problem Area in Diabetes-Pediatric Version Survey and Its Weak Association with Metabolic Control Parameters in Pediatric Diabetes: A Cross-Sectional Multicenter Study
Source: J Clin Med. 2025 Jan 15;14(2):523. doi: 10.3390/jcm14020523 (PMC11765515; doi:10.3390/jcm14020523)
Supplement: Supplementary file 1 [file jcm-14-00523-s001.zip › jcm-3397942-supplementary.pdf]

Appendix 1. STROBE Statement—Checklist of items that should be included in reports of ***cross-sectional studies.***

	Item No	Recommendation	Page No
**Title and abstract**	1	(*a*) Indicate the study's design with a commonly used term in the title or the abstract	1
		(*b*) Provide in the abstract an informative and balanced summary of what was done and what was found	2
		**Introduction**	
Background/rationale	2	Explain the scientific background and rationale for the investigation being reported	3-4
Objectives	3	State specific objectives, including any prespecified hypotheses	4
		**Methods**	
Study design	4	Present key elements of study design early in the paper	4-5
Setting	5	Describe the setting, locations, and relevant dates, including periods of recruitment, exposure, follow-up, and data collection	4-5
Participants	6	(*a*) Give the eligibility criteria, and the sources and methods of selection of participants	4-5
Variables	7	Clearly define all outcomes, exposures, predictors, potential confounders, and effect modifiers. Give diagnostic criteria, if applicable	4-5
Data sources/ measurement	8*	For each variable of interest, give sources of data and details of methods of assessment (measurement). Describe comparability of assessment methods if there is more than one group	4-5
Bias	9	Describe any efforts to address potential sources of bias	4-5
Study size	10	Explain how the study size was arrived at	4-5
Quantitative variables	11	Explain how quantitative variables were handled in the analyses. If applicable, describe which groupings were chosen and why	4-5
Statistical methods	12	(*a*) Describe all statistical methods, including those used to control for confounding	5
		(*b*) Describe any methods used to examine subgroups and interactions	5
		(*c*) Explain how missing data were addressed	/
		(*d*) If applicable, describe analytical methods taking account of sampling strategy	/
		(*e*) Describe any sensitivity analyses	5
		**Results**	
Participants	13*	(a) Report numbers of individuals at each stage of study—eg numbers potentially eligible, examined for eligibility, confirmed eligible, included in the study, completing follow-up, and analysed	8
		(b) Give reasons for non-participation at each stage	8
		(c) Consider use of a flow diagram	7
Descriptive data	14*	(a) Give characteristics of study participants (eg demographic, clinical, social) and information on exposures and potential confounders	11-13
		(b) Indicate number of participants with missing data for each variable of interest	/
Outcome data	15*	Report numbers of outcome events or summary measures	11-13
Main results	16	(*a*) Give unadjusted estimates and, if applicable, confounder-adjusted estimates and their precision (eg, 95% confidence interval). Make clear which confounders were adjusted for and why they were included	16-16
		(*b*) Report category boundaries when continuous variables were categorized	13-15
		(*c*) If relevant, consider translating estimates of relative risk into absolute risk for a meaningful time period	/
Other analyses	17	Report other analyses done—eg analyses of subgroups and interactions, and sensitivity analyses	11-18
		**Discussion**	
Key results	18	Summarise key results with reference to study objectives	18-21
Limitations	19	Discuss limitations of the study, taking into account sources of potential bias or imprecision. Discuss both direction and magnitude of any potential bias	22-23
Interpretation	20	Give a cautious overall interpretation of results considering objectives, limitations, multiplicity of analyses, results from similar studies, and other relevant evidence	23
Generalisability	21	Discuss the generalisability (external validity) of the study results	22-23
		**Other information**	
Funding	22	Give the source of funding and the role of the funders for the present study and, if applicable, for the original study on which the present article is based	24

*Give information separately for exposed and unexposed groups.

Note: An Explanation and Elaboration article discusses each checklist item and gives methodological background and published

examples of transparent reporting. The STROBE checklist is best used in conjunction with this article (freely available on the Web sites of PLoS Medicine at http://www.plosmedicine.org/, Annals of Internal Medicine at http://www.annals.org/, and Epidemiology at http://www.epidem.com/). Information on the STROBE Initiative is available at www.strobe-statement.org.

APPENDIX 2: Aspectos problemáticos de la diabetes – cuestionario pediátrico versión en español (PAID – Peds versión español).

Las siguientes afirmaciones describen cuestiones relacionadas con la diabetes que pueden suponer o no un problema para ti. Elige la respuesta que mejor describa tu grado de acuerdo o desacuerdo con la afirmación de cada punto.

DURANTE EL ÚLTIMO MES…	De acuerdo	→	Ni de acuerdo ni en desacuerdo	→	En desacuerdo
1. Me pongo muy triste cuando pienso en que tengo diabetes.	0	1	2	3	4
2. Siento que la diabetes controla mi vida.	0	1	2	3	4
3. Siento que es mi culpa cuando mi nivel de azúcar está fuera de rango.	0	1	2	3	4
4. Me molesta tener que estar pensando en lo que como.	0	1	2	3	4
5. Me preocupo constantemente por cómo me afectará la diabetes cuando sea mayor.	0	1	2	3	4
6. Me molesta que mi nivel de azúcar esté fuera de rango.	0	1	2	3	4
7. Estoy demasiado cansado/a de tener diabetes como para cuidar de ella.	0	1	2	3	4
8. Me siento excluido/a cuando no puedo comer lo mismo que otros niños/as o adolescentes.	0	1	2	3	4
9. Me fastidia tener que dejar de hacer lo que estoy haciendo para medir mi azúcar en sangre.	0	1	2	3	4
10. Estoy cansado/a de intentar calcular la dosis de insulina en cada comida.	0	1	2	3	4
11. Me avergüenza tener diabetes.	0	1	2	3	4
12. Mis amigos y/o mi familia se comportan como la «policía de la diabetes» (por ejemplo, me recuerdan continuamente que coma bien, que me mida el azúcar en sangre o que me ponga la insulina).	0	1	2	3	4
13. Estoy cansado/a de tener que acordarme de ponerme las inyecciones de insulina o los bolos.	0	1	2	3	4
14. Tengo la sensación de que, haga lo que haga, mis niveles de azúcar siempre están fuera de control.	0	1	2	3	4
15. Siento que no encajo con otros niños/as o adolescentes de mi edad por culpa de la diabetes.	0	1	2	3	4
16. Me molesta tener que rotar las zonas de inyección o de infusión de la bomba.	0	1	2	3	4
17. Me enfado mucho cuando pienso en que tengo diabetes.	0	1	2	3	4
18. Mis amigos y mi familia no entienden lo que es tener diabetes.	0	1	2	3	4
19. Me preocupa tener una bajada de azúcar, especialmente al hacer ejercicio (deportes, juegos al aire libre, clases de baile, etc.).	0	1	2	3	4
20. Mis padres se preocupan demasiado por mí y por mi diabetes.	0	1	2	3	4

Instructions For Obtaining the Score:

Problem Areas in Diabetes Survey – Pediatric version (PAID-PEDS®).

Attributes Of the Survey:

- 20 items.
- Response options: Likert Scale Score from 0 to 4 (0 = Agree; 4 = Disagree).
- Total possible score: 0 to 100.
- Higher scores indicate greater perception of emotional burden.

Instructions

Reverse the score obtained for each item.

Original response	Reversed score
0	4
1	3
2	2
3	1
4	0

Calculate the average of all obtained scores.

Multiply the value by 25 to normalize the total score to a range of 0–100.

How to reference in publications/presentations:

Casanovas-Marsal JO, Civitani Monzón E, Ferrer Duce MP, Ferrer Lozano M, Vara Callau M, González de la Cuesta D, Yelmo Valverde R, Pérez Repiso V, Goicoechea Manterola I, de Arriba Muñoz A. Translation and psychometric evaluation of the Spanish version of the problem areas in diabetes-pediatric version (PAID-Peds®) survey. Diabetol Metab Syndr. 2023 Oct 30;15(1):219. DOI: 10.1186/s13098-023-01199-3.

Original article in English:

Markowitz JT, Volkening LK, Butler DA, Laffel LM. Youth-Perceived Burden of Type 1 Diabetes: Problem Areas in Diabetes Survey-Pediatric Version (PAID-Peds®). J Diabetes Sci Technol. 2015 Apr 24;9(5):1080-5. DOI: 10.1177/1932296815583506.
